# Distribution and trends of cancer in Buffalo City, Eastern Cape Province, 1991-2009: a retrospective study

**DOI:** 10.11604/pamj.2022.42.301.30575

**Published:** 2022-08-22

**Authors:** Nomfuneko Sithole, Themba Geoffrey Ginindza, Nontuthuzelo Iris Muriel Somdyala

**Affiliations:** 1 South African Medical Research Council, Burden of Disease Research Unit, Francie van Zijl Drive, Parow Valley, Cape Town, 7505, South Africa,; 2 Discipline of Public Health Medicine, School of Nursing and Public Health, University of KwaZulu-Natal, South Africa

**Keywords:** Cancer, cancer distribution, cancer trends, age standardised rates, Buffalo City, Eastern Cape Province

## Abstract

Despite interventions, cancer remains a global public health concern. Cancer burden continues to grow globally, demanding the implementation of important preventative and control initiatives. Informative reports on existing routine cancer data are therefore required. This study describes the distribution and trends of cancer in Buffalo City (BFC) population, Eastern Cape (EC) Province for the period 1991-2009. Cancer patients were retrospectively surveyed in the database of Frere Hospital Oncology-Radiation Unit. Proportion by sex, race, geographical distribution in the province and top cancer sites were calculated. Direct method of standardisation was used to calculate Age Standardised Rates (ASR) for a subpopulation of patients residing at BFC; age-specific rates were applied to the reference population (World Standard Population). Trends in rates with 95% Confidence Intervals (CI) for two most common cancers in males and females over time adjusted for age and sex and interactions between time and race were assessed using Poisson Regression. A total of 19 737 patients´ records were analysed; 38.8% (7 656) males and 61.2% (12 081) females. Most patients were Black Africans (81.5%), followed by Whites (13.5%), Mixed Race (4.5%) and Asians (0.5%). A larger proportion (46.0%) of the patients were from Buffalo City, while the rest were distributed in other municipalities served by Frere Hospital. Top five cancers in males were lung [22.5%, ASR 21.0], prostate [14.7%, ASR 9.2], larynx [5.8%, ASR 5.0], mouth [4.4%, ASR 3.7] and tongue [3.8%, ASR 2.9 per 100 000] in females; cervix [20.9%, ASR 23.0], breast [23.6%, ASR 20.2], lung [3.4%, ASR 4.7], ovary [2.1%, ASR 3.0] and corpus uteri [3.4%, ASR 2.8]. Trends showed a decrease in lung and prostate cancers in males, while cervix and breast remained stable in females. White males were two times (95% CI: 1.87-2.49) more likely to have lung cancer and five times (95% CI: 3.90-6.21) more likely to have prostate cancer than Black Africans. White females were 0.43 (95% CI: 0.44-0.73) less likely to have cervical cancer and three times (95% CI: 2.45-3.14) more likely to have breast compared to Black Africans. In conclusion, the availability of routine cancer data at Frere Hospital contributes to strengthening of the national cancer notification regulation, as the results of this study suggest that the burden of cancer in the EC Province remains high.

## Introduction

Despite the implementation of different interventions, cancer remains a global public health concern. Cancer is the second leading cause of death globally, with an estimate of 9.6 million deaths, which means one in six deaths, in 2018 [1, 2]. Lung, prostate, colorectal, stomach and liver cancers are the most common types in men, while breast, colorectal, lung, cervical and thyroid cancers are the most common among women [1, 2]. Approximately 80% of these cancer cases occur in Low and Middle-Income Countries (LMICs) [1, 2]. According to the World Health Organisation (WHO), the cancer burden continues to grow globally, exerting tremendous physical, emotional and financial strain on individuals, families, communities, and health systems [3]. Many health systems in LMICs have limited resources to manage this burden, and such result to many cancer patients who never have access to timely, quality diagnosis and treatment [3].

South Africa (SA) is experiencing a quadruple burden of disease, characterized by a rapid shift from communicable disease domination to increasing non-communicable disease (NCDs) [4]. Better management of infectious disease mortality has resulted in increasing life expectancy, which in turn resulted in a rise in NCDs [5]. Cancer contributes greatly to the observed NCD burden [4]. Based on the most recent SA National Cancer Registry (NCR) report, 1 in 7 women and 1 in 6 men are at risk of developing cancer in their lifetime [6]. The most diagnosed cancers among women are breast, cervix, colorectal, uterus and lung, which together account for half of all female cancers [6]. Among men the reported common cancers are prostate, colorectal, lung, bladder and oesophagus, which together total 37% of all cancers in men [6]. The observed increase in cancer burden is attributable to increasing aging, growth of the population as well as increased prevalence of risk factors associated with economic transition, smoking, obesity, physical inactivity, reproductive behaviors, and westernized types of diet [7]. In urban populations of SA, there have been changes in reproductive factors toward earlier menarche, delayed childbearing, and lower fertility [8]. There is evidence for the rising burden of cancers associated with these risk factors, for example breast cancer age standardised incidence rates increased from 31 to 36 per 100 000 from 2001 to 2016 [6] and nearly 8% (38 000) of the total deaths in SA in 2014 were attributed to cancer [9].

In recognition of the growing burden of cancer in SA, important initiatives to prevent and control cancer have been implemented. These include the amendment of the 1993 Tobacco Products Control Act which restricted the advertising of tobacco products, prohibited smoking in public places and tobacco manufacturer´s sponsorships, the legal minimum age for purchasing tobacco was determined and moved from 16 to 18 [10]. This was a government effort to reduce lung cancer and other upper respiratory tract conditions associated with tobacco smoking. The introduction of a cervical cancer screening programme in 2000 was proposed for screening women over the age of 30 years and offer asymptomatic women 3 free smears in a lifetime, 10 years apart [11]. However, the uptake and impact of this policy on cervical cancer incidence in SA has been poor due to its fragmented and uncoordinated implementation [10, 11]. Additionally, cancer control initiatives include the establishment of a new regulation on cancer registration in April 2011 to enhance cancer reporting, revitalization of the NCR by the SA Department of Health [12]. Cancer registration regulation makes it compulsory for every health facility to record and notify every cancer case that is diagnosed. A Ministerial Advisory Committee on the prevention and control of cancer was also established in year 2013 [10]. However, as imperative as it is to prevent and control cancer and henceforth strengthen cancer surveillance in health facilities, there remains the need to review previous cancer cases where routine patients´ records are available to make the data informative and contribute to future cancer control initiatives.

## Methods

**Aim:** the aim of this study was to describe the distribution and trends of cancer in Buffalo City (BFC) population of the Eastern Cape (EC) Province for the period 1991 to 2009.

### Study design

A retrospective survey was done at Frere Hospital between 1^st^ January 1991 and 31^st^ December 2009 where medical records of cancer patients were retrieved from the Oncology-Radiation Unit database. This was an initial enquiry into what is the burden of cancer at this hospital and in the population of BFC urban area of the EC Province. Since this was a 19-year review, case recurrences and metastasis in the same individuals were excluded. A commonly regarded conclusion was that most cancer cases from BFC area would have been attended to at this referral hospital as it is the only hospital with an oncology specialist and provides oncology services in the region.

### Study area and population

**Figure 1:**
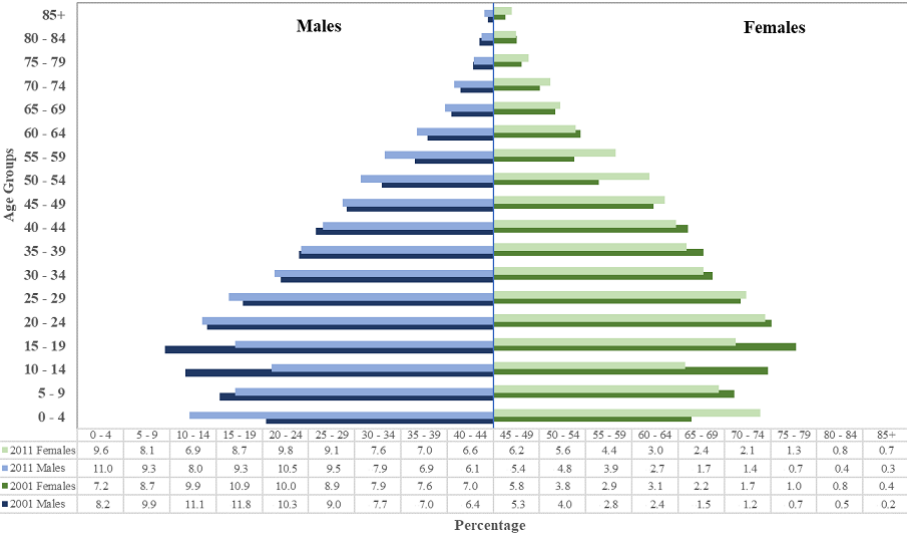
Eastern Cape Province population distribution by age and sex (Statistics South Africa, 2012)

EC Province is the second-largest province in SA. It has the third-largest population, approximately 6.5 million people, which is 12.7% of SA´s population [13]. The demographic profile of residents indicates that the population aged 14 years or below decreased from 36.6% in 2001 to 33.0% in 2011 whilst that between ages 15 and 64 years increased from 57.1% to 60.2% [13] (Figure 1). The proportion of females in the province is 52.9% whilst males are 47.1%. Black Africans constitute the largest group of the population at 86.3%, followed by Mixed Race (8.3%), Whites (4.7%), Asians (0.4%) and the remaining 0.3% are other [13]. There are two major urban areas within the EC Province, namely Nelson Mandela and Buffalo City (BFC) Metropolitan Municipalities. BFC is the key urban area of the eastern part of the province. It consists of several urban areas, including East London. According to census 2011 the size of BFC population is 755 198 [13]. Frere Hospital, situated in East London, forms part of the East London Hospital Complex, which offers specialist medical care to the largest population in the province. The study population was all cancer patients who visited Frere Hospital Oncology-Radiation Unit.

### Data collection

Frere Hospital´s Oncology-Radiation Unit´s database dates to the year 1990. The database is generated by continuous recording of each patient visiting the facility daily. Recording into a Radiotherapy (RT) programme is done by the departmental data clerks using pre-defined variables. Cancer patients in this database were retrieved for a period of 19 years, from 01 January 1991 to 31 December 2009. This was the most up-to-date information at the time of the study. An Information Technology (IT) specialist employed by the South African Medical Research Council assisted with extracting data from the department´s database software (Radiotherapy Software) and transfer it into an excel spread sheet to prevent messing up the database. Mandatory variables that were extracted included patient demographics (first name, surname, sex, age, address, and racial group), tumor information; site and date of diagnosis.

**Data cleaning:** data cleaning incorporated techniques such as checking for completeness and accuracy of patient information details. Mandatory variables were checked if they were available and complete for each patient. Validation checks were carried out on International Classification of Diseases (ICD) codes; some diagnosis codes were in ICD-9 format and these were converted into ICD-10. Data clerks were contacted for clarity on information when needed. Consistency checks were carried out to ensure the concordance of specified data items against other recorded items e.g. prostate cancer in a female or cervical cancer in a male and these were corrected. Dates were formatted into month-day-year and sequence checked so that the date of birth preceded the date of diagnosis and the date of last seen. Age less than zero and greater than ninety-nine was replaced as missing. Geographical areas were coded according to the South African Population Census. Data were further cleaned for inclusion in the analysis. Cases which were excluded from the analysis included duplicate cases, non-malignant cases (e.g. thyrotoxicosis cases), as well as cases with missing diagnosis and age.

**Data analysis:** data were subsequently analysed using STATA 14.0 analysis software to determine the proportion of cases by sex, race, geographical distribution in the province and top 10 cancer sites. The Direct Method of Standardization was used to calculate ASR [14] for the BFC population, which was estimated using the 2007 community survey and 2011 SA Population Census. Age-specific rates for each age group of BFC population, arranged in 10-year age groups, were calculated by dividing the number of cancer cases in the age group by the number of BFC population persons in the age group.


$$Age\text{ specific rate = }\frac{Number_{\text{cancer cases in specific age group}}*100000}{Number_{\text{BFC estimated population persons in specific age group}}}\;(1)$$

ASRs for each cancer site were obtained from the SUMPRODUCT of age specific rates for the cancer site multiplied by 100 000 population and the product was divided by the sum of the World Standard Population (WSP). The WSP of 100 000 is a reference population generally applied to cancer data, to allow for comparison of results with other populations [15].


$$ASR=\frac{\sum SUMPRODUCT{\text{ (R}}_{\text{1}}{\text{P}}_{\text{1}}\text{)}}{\sum sumP_{1}}\text{ (2)}$$

**R1**= is the age specific rate for age group 1 in the population being studied; **P1**= is the population of age group 1 in the standard population.

Poisson's regression analysis, performed in STATA software, was used to assess trends in rates with 95% Confidence Intervals (95% CI) for 2 most common cancers in males and females over time adjusted for age and sex and interactions between time and race.

### Ethical considerations

Ethics approval was obtained from the University of the Western Cape Ethics Committee (REG. NO. 13/2/26) before commencement of the study. Furthermore, permission was obtained from the Director of Clinical Governance of Frere Hospital and the Research Committee of the Eastern Cape Province to access patient records and perform the study. Careful measures on confidentiality regarding patient information were exercised. After the exclusion of duplicate cases, patient personal details were replaced with special numbers for each patient.

## Results

A total of 20 350 cancer cases were recorded in Frere Hospital Oncology-Radiation Unit for the period 1991-2009, with an annual average of 1 071. Only 19 737 (97%) cases, with cancer and had all variables, were included in the analysis (3% excluded due to missing information). A consistent number of cases was observed with a noticeable increase from 2003 (n=959) to 2007 (n=1332). However, a 5.6% decline in number of cases in 2008 was noticed.

### Characteristics of cancer cases seen at Frere Hospital

#### Sex and race

Females accounted for 61.2% (12 081) of the cancer cases and males 38.8% (7 656) of the total cases recorded during this period. The percentage of male cases dropped from 41.2% in 1991 to 28.7% in 2009 while female cases increased from 58.8% to 71.2%. The majority (81.5%) of the cancer cases were Black Africans followed by Whites with 13.5%, Mixed Race with 4.5% and Asians with 0.5%. The number of cancer cases increased among Black African as compared to Whites: 74.3% in 1991 to 89.9% in 2009 vs. 24.3% to 8.0%. The total number of cancer patients with medical aid information dropped to zero by year 2009.

### Geographical distribution of cancers cases seen at Frere Hospital

**Figure 2:**
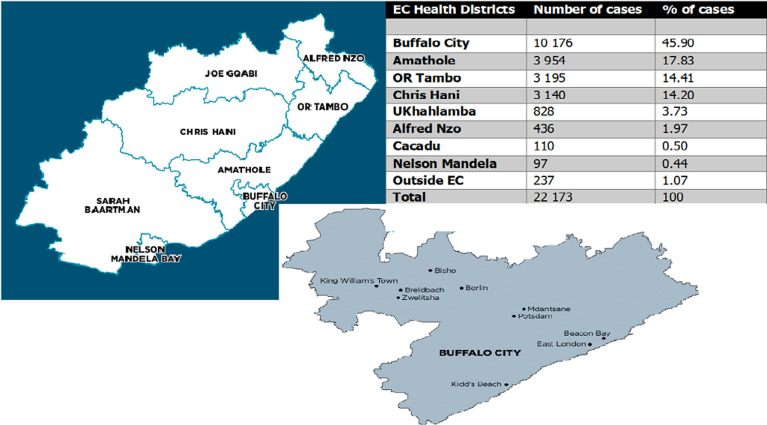
percentage distribution of cases across municipalities in the Eastern Cape Province, 1991-2009

A large proportion of 46.0% cases were from Buffalo City Local Municipality while the rest were distributed around municipalities served by Frere Hospital Oncology-Radiation Unit, which include Amathole (18.0%), OR Tambo (14.0%), Chris Hani (14.0%), UKhahlamba (4.0%), Alfred Nzo (2.0%), Cacadu (0.5%) and Nelson Mandela (0.4%) (Figure 2). About 1.1% were from outside the Eastern Cape Province.

### Most common cancers seen at Frere Hospital

Overall top 10 cancers recorded include cervix (22.4%), breast (14.0%), lung (9.4%), mouth (4.3%), colon (3.9%), larynx (3.7%), prostate (2.8%), tongue (2.5%), oesophagus (2.2%) and Non-Hodgkin´s lymphoma (2.2%). The top five cancers in males were lung (18.6%), larynx (8.3%), mouth (7.8%), prostate (7.1%), and Tongue (5.2%) whereas in females were cervix (36.6%), breast (22.0%), lung (3.5%), ovary (2.8%) and corpus uteri (2.7%). Unknown primary and ill-defined cancer sites in both males and females featured in the most common cancers´ list; 2.9% and 1.2%, respectively.

### Childhood cancers seen at Frere Hospital

A total of 360 cases were childhood cancers (boys and girls aged 0-14 years) which constituted 1.8% of the total cancers observed during the period. Distribution of cases according to sex was as follows; among boys: brain (21.1%), nephroblastoma (18.7%), retinoblastoma (14.8%), leukemia (9.6%), Hodgkin´s lymphoma (8.1%), bone (8.1%), Non-Hodgkin´s lymphoma (7.7%), testis (5.7%), connective and soft tissue (4.3%) among girls: brain (20.4%), nephroblastoma (17.1%), retinoblastoma (13.8%), bone (12.7%), leukemia (9.9%), connective and soft tissue (7.7%), unknown primary (6.6%), Non-Hodgkin´s lymphoma (6.1%) and Hodgkin´s lymphoma (5.5%).

### Estimated Age Standardised Rates (ASRs) for Buffalo City subpopulation

The overall ASR for males was 83.2 per 100 000 population and for females 83.3 per 100 000 population (Annex 1). Age distribution of cancers showed a peak increase from age 20 years onwards. Table 1 shows the ranking of the top-10 cancer sites by sex. In males the top-5 cancer sites were lung [22.5%, ASR: 21.0 per 100 000], prostate [14.7%, ASR: 9.2 per 100 000], larynx [5.8%, ASR 5.0 per 100 000], mouth [4.4%, ASR: 3.7 per 100 000] and tongue [3.8%, ASR: 2.9 per 100 000] whilst in females were cervix [20.9%, ASR: 23.0 per 100 000], breast [23.6%, ASR: 20.2 per 100 000], lung [3.4%, ASR: 4.7 per 100 000], ovary [2.1%, ASR: 3.0 per 100 000] and corpus uteri [3.4%, ASR: 2.8 per 100 000].

**Table 1:** estimated BFC top 10 cancers by sex and cancer site, 1991-2009

	Males					Females			
Cancer Site	Total	%	CR	ASR	Cancer Site	Total	%	CR	ASR
Lung	379	22.5	14.5	21.0	Cervix	383	20.9	20.7	23.0
Prostate	249	14.7	5.6	9.2	Breast	352	23.6	18.4	20.2
Larynx	98	5.8	3.4	5.0	Lung	83	3.4	3.9	4.7
Mouth	74	4.4	2.6	3.7	Ovary	46	2.1	2.7	3.0
Tongue	64	3.8	2.0	2.9	Corpus uteri	63	3.4	2.3	2.8
Unknown Primary	56	3.3	2.1	2.9	Colon	43	2.4	2.1	2.3
Colon	52	3.1	2.1	2.9	NHL	34	1.7	1.9	2.0
Oesophagus	43	2.5	1.9	2.6	Mouth	47	6.2	1.5	1.7
Rectum	49	2.9	1.5	2.3	Oesophagus	27	1.4	1.4	1.6
NHL	41	2.4	1.8	2.2	Multiple Myeloma	30	2.1	1.4	1.6
Multiple Myeloma	50	2.9	1.5	2.2	Unknown Primary	96	1.9	1.4	1.6
Remaining sites	532	31.7	20.7	24.2	Remaining sites	296	30.9	17.6	18.8
**Total**	**1 155**	**68.3**	**38.2**	**59.0**	**Total**	**1 204**	**69.1**	**57.7**	**64.5**

NHL - Non-Hodgkin Leukemia

### Cancer trends and differentials

**Figure 3:**
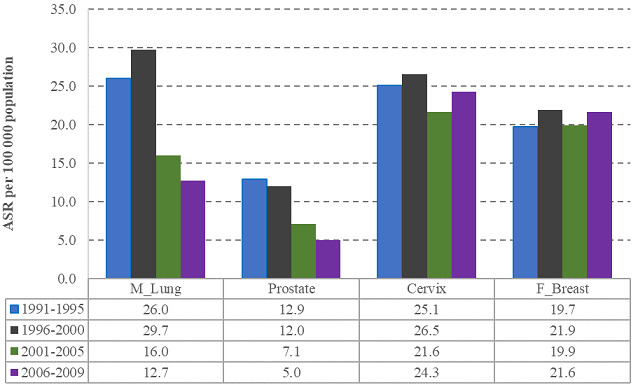
trends in selected cancer sites in males and females, 1991-2009

**Figure 4:**
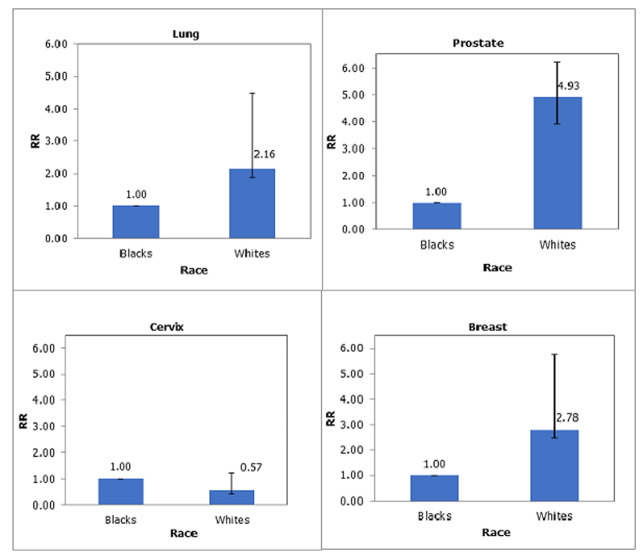
top 2 cancer rate ratios in males (top) and females (bottom) by race, 1991-2009

**Figure 5:**
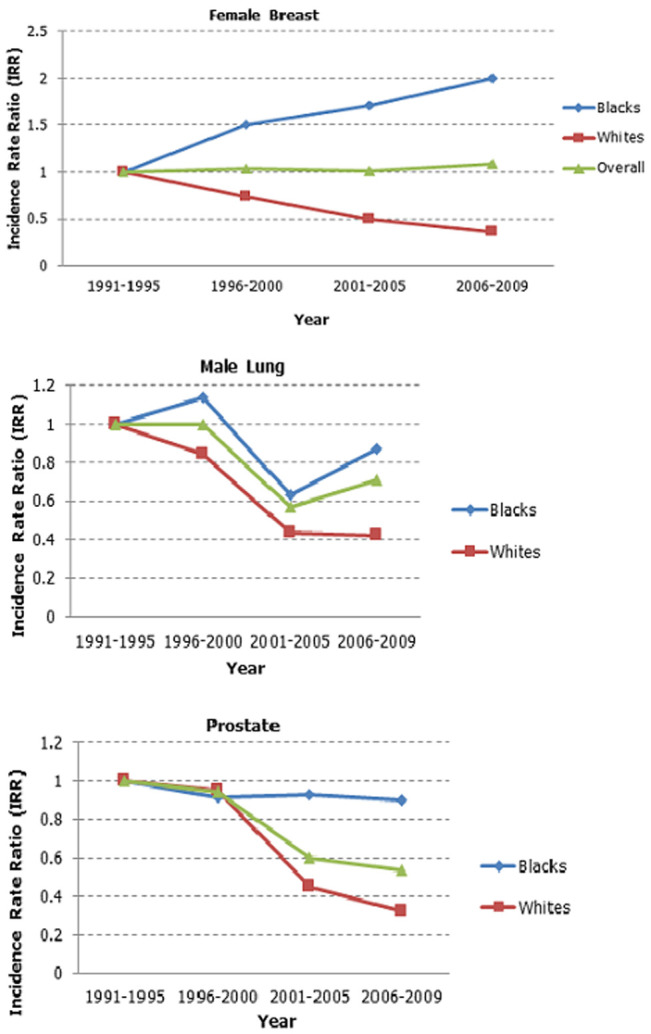
time and race interaction for female breast, male lung and prostate cancers, 1991-2009

Trends of top two cancer sites for males and females are shown in 5-year periods in Figure 3; male lung cancer ASRs decreased from 26.0 in 1991-1995 to 12.7 in 2006-2009, while prostate cancer decreased from 12.9 to 5.0 in the same period. Cervix (25.1 and 24.3) and female breast (19.7 and 21.6) cancers remained stable. Females were 0.63 (95% CI: 0.32-0.42) less likely to have lung cancer compared to males. Table 2 illustrates that White males were two times (95% CI: 1.87-2.49) more likely to have lung cancer and five times (95% CI: 3.90-6.21) more likely to have prostate cancer than Black Africans. While White females, were 0.43 (95% CI: 0.44-0.73) less likely to have cervical cancer and three times (95% CI: 2.45-3.14) more likely to have breast cancer compared to Black African females (Figure 4). Figure 5 shows time and race interaction between Black African and White patients for male lung cancer cases, prostate in males and breast cancer in females. While white male cancer rates contributed significantly to the progressive decrease observed over the period. Prostate cancer in Black African males remained stable, and breast cancer incidence rates of Black African females increased while those of White females decreased.

**Table 2:** cancer rate ratio (RR) and confidence interval (CI) by sex and race for top 2 cancer sites, 1991-2009

	Cancer site	Blacks	Whites	CI
		RR	RR	
**Males**	Lung	1.00	2.16	1.87-2.49
	Prostate	1.00	4.93	3.90-6.21
**Females**	Cervix	1.00	0.57	0.44-0.73
	Breast	1.00	2.78	2.45-3.14

## Discussion

The annual number of malignant cases at Oncology Radiation Unit, Frere Hospital increased from 953 in 1991 to 1 414 in 2009. More than half of the malignant cases were observed in females (61.2%). This pattern is reported as unique to the African continent, due, in part, to the large cervical cancer burden [16]. Most patients with cancer were Black African and showed an overall increase from 1991 to 2009 whereas the White population decreased. The high number of Black African patients was expected as Black Africans form most of the Eastern Cape Province population and rural-urban migration is the most common form of internal migration in South Africa [17] even though it was not explored fully in this study. The annual number of patients with medical aid information decreased from 144 in 1999 to zero in 2009. The medical aid information available in the data was thought to be unreliable, even though it probably indicated a trend with fewer private sector patients at Frere Hospital. Cancers were presented by sites, but unknown primary and ill-defined cancers were included in the analysis, with results showing considerably high numbers in both males and females. South Africa is a middle-income country where open-access tertiary hospitals such as Frere Hospital can provide early detection and care to lessen the burden of cancer. However, deep-rooted limitations exist which prevent access resulting to late presentation (stages III and/or IV) and diagnosis, thus the high percentage of unknown primary and ill-defined cancers observed in this study [18, 19]. Compared with those observed for the period 1952 to 1956 reported by Burrel in 1957 the overall leading cancers in this study differed (Annex 2). Burrel, reported oesophageal cancer as the leading cancer and lung cancer did not feature [20]. The absence of oesophageal cancer in the top three cancers in this data is also striking when compared with contemporary studies in the Eastern Cape Province as well as in Eastern and Southern Africa Sub-regions [21-25]. In the Sub-Saharan African Region, oesophageal cancer is reported to be commonly diagnosed at late stages, with poor prognosis and limited treatment. This leads to a high case fatality rate despite some countries having the state-of-the-art specialized cancer centres [26, 27]. It is therefore, possible that some oesophagus cases were missing in this study as they were not referred for medical care in this hospital.

Our findings showed that lung (22.5%, ASR: 21.0), prostate (14.7%, ASR: 9.2), cervix (20.9%, ASR: 23.0) and female breast (23.6%, ASR: 20.2) were top two leading cancers in males and females respectively. These cancers are reported to be the most commonly diagnosed cancers and contribute greatly to the increasing burden of cancer in Africa [1, 5, 28]. Lung cancer in males showed a decreasing trend over the period of observation. This could be expected as South Africa is reported to have been a leader in development and implementation of appropriate tobacco control plans. This has resulted in a national decrease in prevalence of smoking amongst adults as well as in learners (from 23% in 1999 to 16.9% in 2011) [7, 29]. Chokunonga, *et al*. in 2013 reported a decrease in lung cancer incidence rates of the studied population from 1991 through 2010 [30]. Prostate cancer in South Africa is reported to have the highest incidence (40.5 per 100 000) compared to other African countries [31]. Observed high prostate cancer rates in this study could be reflective of enhanced diagnostic capabilities, notably increased availability of Prostate Specific Antigen (PSA) testing at this urban hospital. Furthermore, White males contributed significantly to the overall decrease in trend of prostate cancer cases over the period.

Reproductive cancers; cervix and breast were the most common cancers observed in this study which compares to a study by Parkin *et al*. [28] accounting for 20.4% and 27.6%, respectively. The incidence of cervical cancer was reported to be highest in Eastern and Southern Africa (30-40 per 100 000) whilst that of breast cancer (35 per 100 000) was highest in South Africa [28]. The natural history of cervical cancer has been studied, and it has been concluded that the persistent infection of the cervix by the oncogenic types of *Human papillomavirus* (HPV 16 & HPV 18) is the necessary cause in 72% of cervical cancers in Africa [11, 31]. South Africa is reported to be home to the largest number of HIV-infected individuals, with nearly 60% of incident HIV infections occurring in women [32-34]. Previously performed systematic reviews and analysis have showed that the overall risk of HIV acquisition in women doubled when they had a prevalent HPV infection with any genotype, and that HIV acquisition was significantly associated with any HPV infection [35-37]. Therefore, high numbers of cervix cancer cases in this study could be related to HIV or HPV or a combination of these infections. Further studies are recommended since none of the HIV defining cancers; Kaposi sarcoma or Non-Hodgkin Lymphoma were observed in the top 10 cancers of females in this study. South African women in general are reported to have the highest incidence rates of breast cancer compared to other African regions [28]. This cancer is reported to be higher in White female population compared to Black African population [38, 39]. In South Africa, risk factors include high prevalence of reproductive issues such as early menarche and late child bearing in white females and vice versa with black African [40]. Other risk factors for breast cancer are reported to be related to menstrual and reproductive factors, high body mass index, high alcohol consumption, physical inactivity and exposure to exogenous hormones either as contraceptives or as postmenopausal hormon replacement therapy [39]. Even though breast cancer trends over the 19-year period were observed to be stable, the interaction between race and time showed that Black African female cases actually increased over the period whilst White females decreased. In agreement with a report on incidence of breast cancer in South Africa [41], in this study White females were three times more likely to have breast cancer compared to Black African females.

### Strengths and limitations

Frere Hospital offers specialist medical care to the largest population in the EC Province as it is a major referral hospital for patients from less resourced areas of the province and several districts around Buffalo City. To our knowledge, this study represents the first study on distribution and trends of cancer in an urban population of the province. The cancer information of patients screened and diagnosed at this tertiary level hospital over a period of 19 years was analysed. Some information was inaccurate and incomplete; however, that was only 3%. It is important to mention that patients seen in private oncology hospitals were not included in this study resulting to underestimation of the burden of cancer in this population. Despite these limitations, this initial analysis provided useful descriptive background information on recorded cancer patients treated at Frere Hospital Radiation-Oncology Unit.

### Recommendations

Follow-up studies of distribution and trends of cancer in Buffalo City Urban area diagnosed at all cancer patients treating hospitals; including public and private hospitals from year 2010 onwards are recommended. The sample will ensure completeness and representativeness. The South African Guideline on Cancer Registration must be used to access data at all facilities and confidentiality measures must be observed.

## Conclusion

This is the first review and analysis of data generated by Frere Hospital Oncology-Radiation Unit. This study was useful in describing cancer characteristics of patients seen at Frere Hospital as well as estimating cancer incidence rates in an urban population of the Eastern Cape Province. Observed trends in leading cancers in both males and females and the noted population differences give direction to planning and monitoring of intervention initiatives for these cancers, to detect new cancers and changes in existing patterns. Available hospital routine cancer data, such as one maintained by Frere Hospital, are the primary source of information for population-based cancer registries and are urgently needed in other hospitals in this country for better cancer control programmes and evaluation. Furthermore, the availability of routine cancer data at Frere Hospital contributes to strengthening of the national cancer notification regulation.

### What is known about this topic

- The National Cancer Registry (NCR) annually reports on pathology-based only incident cancer cases in South Africa, covering all nine provinces;
- The Eastern Cape Cancer Registry is a rural population-based cancer registry also providing 5-year period reports on cancer incidence in the province; trends during the period 1998-2012 show that oesophagus cancer is the leading cancer in males and second leading cancer in females with a significant decrease in trends observed over the period;
- Cervix cancer in females and prostate cancer are observed to be significantly increasing over the period, assuming a more urban profile of these cancers; no urban population reports are available for cancer incidence cases in the province.

### What this study adds

- This is the first study of cancer incidence in an urban population of the Eastern Cape Province of South Africa;
- The study does not only estimate cancer incidence but reports on the distribution of cancer cases as well as trends over a 19-year period as an effort to establish base line cancer information of this urban population;
- The study will enable future studies on urban-rural comparison and follow-up of cancer trends in the province as well as identify gaps such as missing information in the hospital-based registry.

## Annexes

Annex 1: estimated BFC minimal cancer incidence rates by cancer site, age group and sex, 1991-2009

Annex 2: a comparison of leading cancer sites at Frere Hospital for the periods (a) 1952-1956 and (b) 1991-2009
